# Pulmonary tumor diagnosed as an undifferentiated sarcoma with epithelioid features: a case report

**DOI:** 10.1186/s13256-016-1056-7

**Published:** 2016-10-03

**Authors:** Mohamed Réda El Ochi, Mohammed Massine El Hammoumi, Abdelhamid Biyi, Mohamed Allaoui, El Hassane Kabiri, Abderrahman Albouzidi, Mohamed Oukabli

**Affiliations:** 1 Department of Pathology, Mohamed V Military Hospital, Hay Riad, Faculty of Medicine and Pharmacy, Mohammed V University, Rabat, Morocco; 2 Department of Thoracic Surgery, Mohamed V Military Hospital, Hay Riad, Faculty of Medicine and Pharmacy, Mohammed V University, Rabat, Morocco; 3 Department of Nuclear Medicine, Mohamed V Military Hospital, Hay Riad, Faculty of Medicine and Pharmacy, Mohammed V University, Rabat, Morocco

**Keywords:** Undifferentiated, Epithelioid, Sarcoma, Lung

## Abstract

**Background:**

Pulmonary sarcomas are uncommon accounting for 0.5 % of all primary lung cancers. Undifferentiated sarcomas account for up to 20 % of soft tissue sarcomas. A lung tumor revealed to be an undifferentiated sarcoma with epithelioid features has never been reported in the literature.

**Case presentation:**

A 61-year-old white Moroccan man presented with 2 months’ history of hemoptysis and dyspnea. Chest computed tomography showed a cystic mass involving the lower field of his right lung evoking first a hydatid cyst. Abdominal computed tomography revealed bilateral adrenal nodules. Surgical resection of the lung mass was performed. On pathological examination, the tumor was cystic containing necrotic material. A histological diagnosis of undifferentiated sarcoma with epithelioid features was made. A positron emission tomography scan showed involvement of his pleura, left colon, adrenal glands, left thigh muscle, and leptomeninges.

**Conclusions:**

Undifferentiated sarcoma with epithelioid features is a rare malignant mesenchymal tumor. Clinical and radiological features are not specific. A differential diagnosis includes sarcomatoid carcinoma, malignant mesothelioma, melanoma, and other epithelioid sarcomas.

## Background

Pulmonary sarcomas are uncommon accounting for 0.5 % of all primary lung cancers. Undifferentiated sarcomas (US) are a heterogeneous group of malignant mesenchymal tumors that do not meet criteria for a well-defined histopathologic entity [1, 2]. They account for up to 20 % of soft tissue sarcomas and occur at all ages and anatomic sites with no difference between the sexes [1]. Clinical and radiological features are not specific. On morphologic examination, US is divided into pleomorphic, spindle cell, round cell, and epithelioid subsets [1]. Cases of US with epithelioid features (USEF) are rarely reported in the literature [3]. We describe the first case of a lung tumor revealed to be a USEF.

## Case presentation

### Clinical history

A 61-year-old white Moroccan man with no significant past medical history presented to Mohamed V Military Hospital with hemoptysis and dyspnea which developed 2 months before admission.

### Radiologic and histopathologic findings

**Fig. 1:**
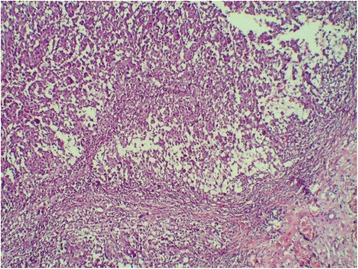
Cystic wall harboring sheets of tumoral cells. Hematoxylin and eosin stain, original magnification ×100

**Fig. 2:**
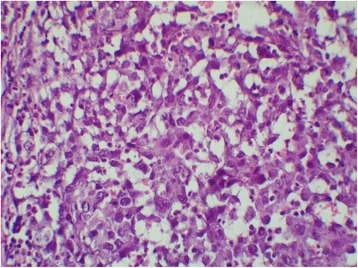
Tumor cells showing epithelioid features and mild nuclear atypia. Hematoxylin and eosin stain, original magnification ×400

**Fig. 3:**
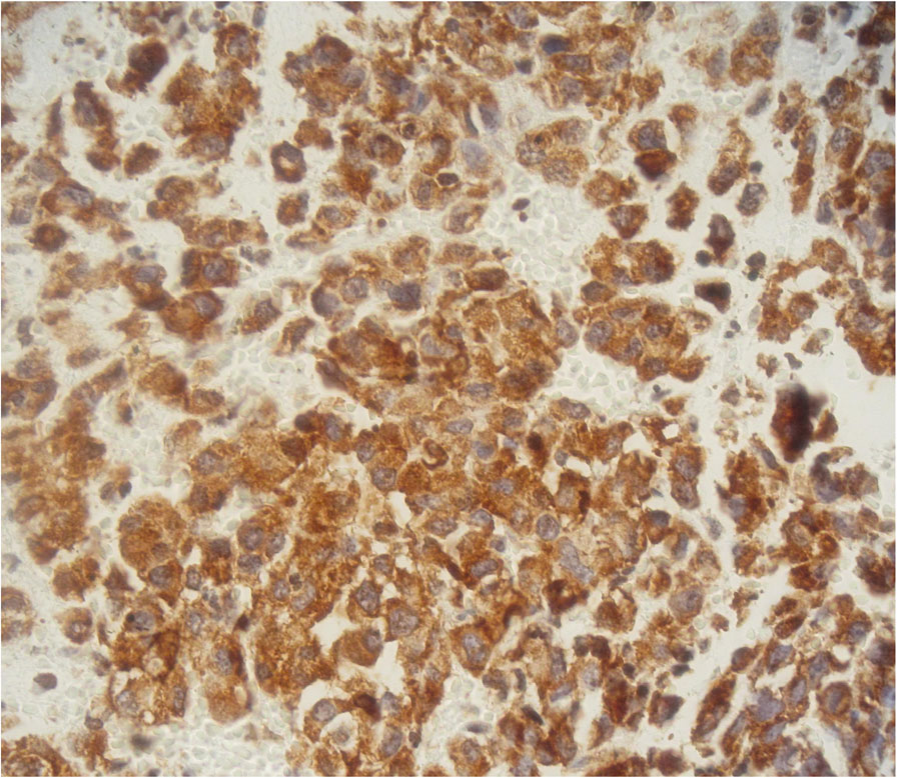
Smooth muscle actin positivity of the tumor cells. Original magnification ×400

Chest computed tomography (CT) showed a cystic mass involving the lower field of his right lung, measuring 5×4.8 cm without mediastinal adenopathy, evoking first a hydatid cyst. Abdominal CT showed bilateral adrenal nodules compatible with nodular hyperplasia measuring 5.3×4.1 cm on the right side and 3×2.6 cm on the left side. Surgical resection of his lung tumor was performed. His postoperative course was uneventful. On pathological examination, the tumor was cystic and contained necrotic material. A histological examination showed nests and sheets of epithelioid cells (Fig. 1). The cells had an abundant and amphophilic cytoplasm (Fig. 2). The nuclei were vesicular and mitoses were numerous (27 mitoses per 10 high-power fields). The tumor cells displayed diffuse immunoreactivity for vimentin and smooth muscle actin (Fig. 3). CD99 was focally positive. Cytokeratin (CK) AE1/AE3, epithelial membrane antigen (EMA), CK7, CK20, P63, CK34BE12, CK5/6, TTF1, calretinin, WT1, D2-40, desmin, myogenin, H-caldesmon, S100 protein, INI 1, melan A, HMB45, CD34, CD31, MDM2, CD117, DOG1, CD20, CD3, CD30, and placental alkaline phosphatase (PLAP) were all negative. Thus, a diagnosis of USEF grade III FNCLCC (La Fédération Nationale des Centres de Lutte Contre le Cancer; The National Federation of Centers of Cancer Control) was made. The surgical margins were positive. His postoperative course was uneventful.

**Fig. 4:**
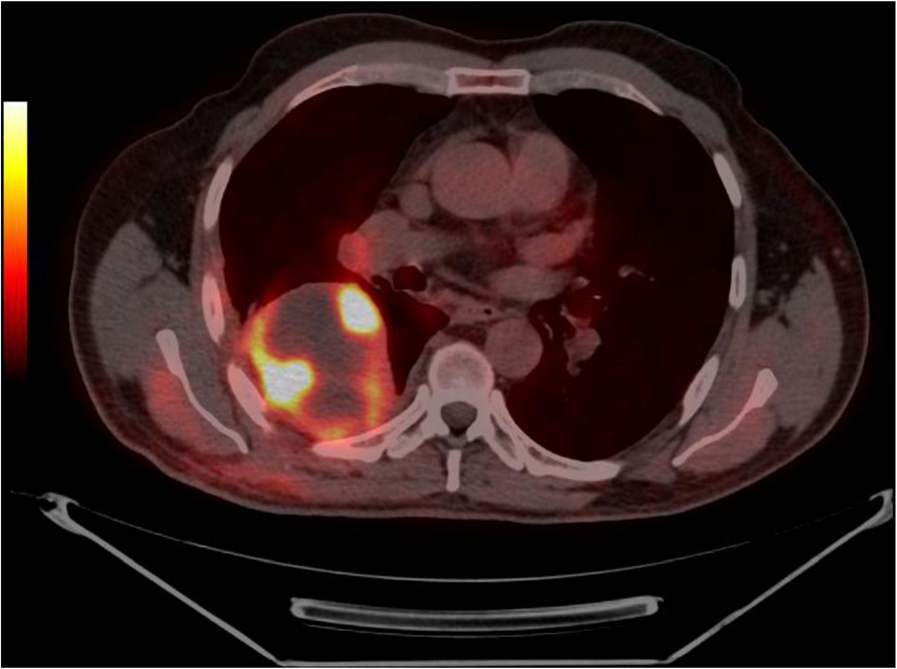
Axial ^18^F-fluorodeoxyglucose positron emission tomography/computed tomography fused image showing increased fluorodeoxyglucose uptake in the lung tumor

**Fig. 5:**
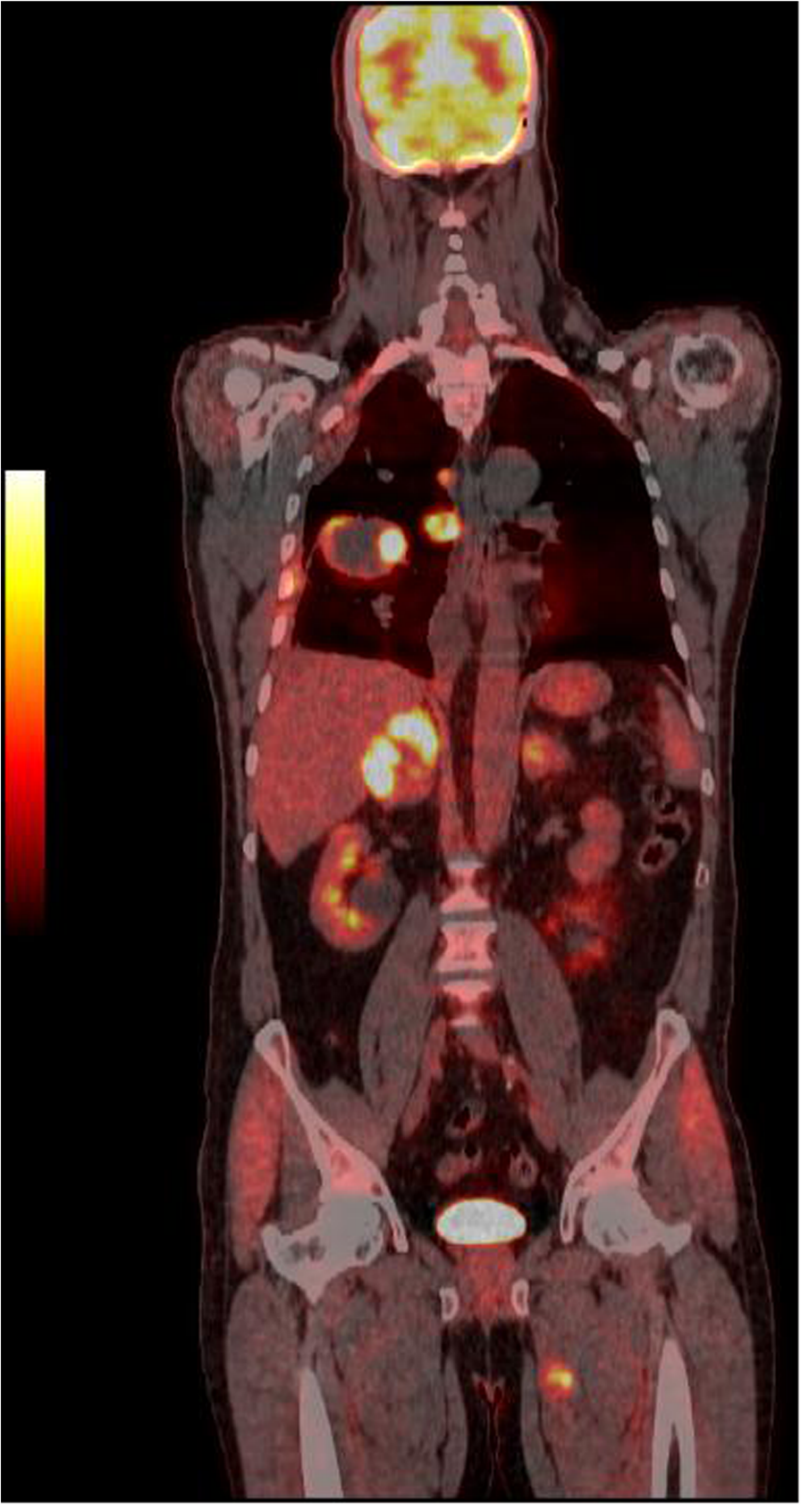
Coronal 18F-fluorodeoxyglucose positron emission tomography/computed tomography fused image showing hypermetabolic foci in the right lung, mediastinal lymph node, adrenal glands and the left thigh

A positron emission tomography (PET) scan, performed subsequently on the 28th day after surgery, visualized foci of increased ^18^F-fluorodeoxyglucose (^18^F-FDG) uptake in his lung tumor (Fig. 4), left colon, adrenal glands, left thigh muscle (Fig. 5), and leptomeninges. A colonoscopy was normal. He is undergoing anti-mitotic chemotherapy.

## Discussion

US are a heterogeneous group of malignant mesenchymal tumors that do not meet criteria for a well-defined histopathologic entity [1, 2]. It is a diagnosis of exclusion that accounts for 20 % of sarcomas [1]. US occur at all ages and anatomic sites with no difference between the sexes [1, 3]. Most reported cases are pleomorphic. The epithelioid variant has been rarely reported. Sarcomas of the lung are mainly metastatic [4]; primitive pulmonary sarcoma are very uncommon and comprise 0.5 % of all primary lung cancers [5].

Clinical and radiological features of sarcoma are not specific and sometimes asymptomatic [1, 6]. The macroscopic findings are not distinctive but tumoral necrosis is frequent [1]. On morphologic examination, USEF is composed of nests of cells with amphophilic cytoplasm and large vesicular nuclei [1]. USEF lacks specific immunohistochemical abnormalities; tests for vimentin are positive and tests for smooth muscle actin are sometimes positive [7]. Tests for CKs, desmin, EMA, CD99, and CD34 are generally negative [1]. The differential diagnosis includes sarcomatoid carcinoma, malignant mesothelioma, melanoma, and other sarcomas such us epithelioid sarcoma, leiomyosarcoma, rhabdomyosarcoma, angiosarcoma, epithelioid hemangioendothelioma, dedifferentiated liposarcoma, synovial sarcoma, and metastatic epithelioid gastrointestinal stromal tumor [1, 4, 6].

The treatment of USEF is similar to epithelioid sarcoma [3]. In localized form, the treatment is based on surgical excision followed by adjuvant doxorubicin-based chemotherapy and sometimes radiotherapy [3]. The treatment of metastasizing tumors is based mainly on chemotherapy. The prognosis of USEF is poor and the 5-year survival is 52 % [3]. Recurrences and metastases occur in 25 % and 35 % of cases respectively [3].

## Conclusions

In summary, USEFs are extremely rare malignant sarcomas. They can occur at all ages and anatomic sites. The epithelioid variant has been rarely reported. The diagnosis is based on morphological and immunohistochemical analyses that allow exclusion of differential diagnoses. In our case, the location posed additional diagnostic difficulties.

## Abbreviations

CK: Cytokeratin
CT: Computed tomography
EMA: Epithelial membrane antigen
FDG: Fluorodeoxyglucose
PET: Positron emission tomography
PLAP: Placental alkaline phosphatase
US: Undifferentiated sarcoma
USEF: Undifferentiated sarcoma with epithelioid features
